# A novel modified flapless surgical technique for sutureless scleral fixation of FIL SSF intraocular lens: a prospective study

**DOI:** 10.1007/s00417-025-07055-6

**Published:** 2025-12-22

**Authors:** Danilo Iannetta, Marc D de Smet, Simone Febbraro, Nicola Valsecchi, Antonio Moramarco, Oscar Matteo Gagliardi, Lorenzo Motta, Luigi Fontana, Alessandro Lambiase

**Affiliations:** 1 Department of Organs of Sense, University of Rome La Sapienza, Rome, Piazzale Aldo Moro 5, 00185 Italy; 2 Micro Invasive Ocular Surgery Center, Helvetia Retina Associates, Av du Léman 32, Lausanne, Switzerland; 3 Dipartimento di Scienze Mediche e Chirurgiche, Ophthalmology Unit, Alma Mater Studiorum University of Bologna, Bologna, Italy; 4 IRCCS Azienda Ospedaliero-Universitaria di Bologna, Bologna, Italy; 5 Department of Neuroscience, Eye Unit, University of Padua, Padua, Italy

**Keywords:** Sutureless scleral fixation, Secondary lens implantation, Aphakia, FIL SSF intraocular lens, surgical technique

## Abstract

**Purpose:**

To evaluate the anatomical and functional outcomes of sutureless scleral fixation of FIL SSF intraocular lens (FIL SSF IOL- Soleko S.P.A. Pontecorvo, Italy) using a novel modified flapless surgical technique.

**Methods:**

In this prospective study, patients older than 18 years with IOL-bag complex subluxation and a minimum follow-up of 24 months were included. At 2.5 mm from the limbus, two circumferential partial-thickness scleral grooves, 4 mm in length, were sculpted at 3 and 9 o’clock with a 300 microns pre-calibrated knife. According to two different variants of the same technique – trocarless or trocar-assisted − 25-G needles or 25-G trocars were respectively used to perforate the deep scleral grooves at the center. The FIL SSF IOL was injected through a 2.4 mm-wide corneal tunnel, and the T-shaped harpoons were gently driven out of the eye through the scleral holes and grooves. No scleral sutures were placed.

**Results:**

A total of 54 eyes of 54 patients were included. The mean age was 74.0 ± 14.2 years (range 31–96), and 40.7% were female. The mean intra-operative time was 79.9 ± 23.2 min. After 24 months from surgery (mean follow-up 25 ± 1 months), BCVA significantly improved from 0.9 ± 0.9 to 0.4 ± 0.5 LogMAR (*p* < 0.001). The mean IOP showed a downward trend from 20.1 to 17.0 mmHg, although this difference did not reach statistical significance (*p* = 0.083). Overall, 87.3% of patients did not present any complications after 24 months from surgery.

**Conclusion:**

This novel flapless technique for sutureless FIL SSF IOL implantation appears safe, and it may represent a valuable alternative to existing methods, offering reduced tissue manipulation, faster surgical time, and a potentially shorter learning curve. It combines the unique features of this IOL design with minimal tissue manipulation, low risk, a shorter learning curve, and reduced operative time.

**Supplementary Information:**

The online version contains supplementary material available at 10.1007/s00417-025-07055-6.

## Abstract (highlights)

**Key messages:**

***What is known*:**

- Sutureless scleral fixation of intraocular lenses is a standard surgical option in eyes with insufficient capsular support.
- Existing techniques often require challenging steps such as scleral flap creation, haptic cauterization, or off-label use of three-piece IOLs, raising concerns about reproducibility and long-term safety.

***What is new*:**

- We describe a novel technique for sutureless scleral fixation of FIL SSF IOLs, which may eliminate the need for scleral flaps, haptic cauterization, or off-label devices.
- The approach demonstrated significant visual improvement and a low complication rate over a 24-month follow-up.
- This surgical technique could offer a simplified and reproducible alternative to existing techniques, with a favorable safety and efficacy profile.

**Supplementary Information:**

The online version contains supplementary material available at 10.1007/s00417-025-07055-6.

## Introduction

Cataracts are a major cause of reversible vision impairment, the leading cause of blindness worldwide [1, 2], and surgery is the current best option for their management [3, 4]. The modern standard of care consists of phacoemulsification and contemporary intraocular lens (IOL) implantation within the capsular bag - referred to as “primary implantation” [1, 5]. An intact capsular bag and zonular apparatus provide the best support for the lens to guarantee its long-term stability and positioning. Unfortunately, several situations may prevent in-the-bag IOL implantation [6]. These include hereditary and acquired conditions associated with lens subluxation, loss of capsular and/or zonular integrity. Furthermore, the inadequacy of capsular support could be related to a complicated cataract surgery, which may have led to large posterior capsular rupture, zonular dialysis, or bag dialysis.

Given these challenging scenarios, IOL implantation is sometimes deferred to a later surgical time: this approach is called “secondary implantation.” Several alternative solutions have been proposed over the years to address such issues, involving the use of different IOL types to correct aphakia, such as anterior chamber IOLs (AC-IOLs), iris-fixated IOLs (IF-IOLs), and scleral-fixated posterior chamber IOLs (SF-IOLs).

Despite each technique presents distinct advantages, none is free from complications and disadvantages [7].

Implanting AC-IOLs could be technically easy, but these lenses are contraindicated when the iris diaphragm is not intact, as in some traumatized eyes. Contact with anterior segment structures could cause many complications, such as corneal endothelial damage, uveitis, and trabecular meshwork damage [8].

Iris-fixated IOLs do not get in contact with angle structures, avoiding related complications, but a compromised iris diaphragm is a contraindication also for these IOL types, and their implantation may result in iris damage [9].

Scleral-fixated intraocular lenses (SF-IOLs) are placed in the posterior chamber, so they are closer to the nodal point of the eye – a more physiological position for the lens – and more distant from anterior segment structures.

The first described techniques involved the use of 10 − 0 Polypropylene or Gore-Tex-like material for sutured scleral fixation of IOLs through the ciliary sulcus [10]. Concerns arose about potential complications related to the sutures themselves and to the surgical manipulation of the ocular tissues. Subsequently, sutureless scleral fixation procedures have been introduced to reduce such risks and have progressively gained more popularity [11–14].

Agarwal et al. [12] reported a sutureless procedure in which the IOL’s haptics were embedded in scleral tunnels underneath scleral flaps, which were finally closed with tissue glue.

Another innovative technique was introduced in 2017 by Yamane et al. [14], who proposed cauterization of the distal end of the externalized haptics to form an end-bulb flange via a transconjunctival approach. Among the common drawbacks were the off-label use of different 3-piece IOL models, which were not intended for intrascleral fixation, and the risks of IOL tilting and of haptic damage that could result from the surgical manipulation and the trans-scleral passage of the haptics [15].

In recent years, an increasing attention has been put on the FIL SSF IOL (Soleko S.P.A. Pontecorvo, Italy), a hydrophilic acrylic intraocular lens (previously known as the Carlevale lens) designed explicitly for scleral fixation without the need for sutures, whose peculiar arrangement allows a firm anchoring to the sclera thanks to the flexible sclero-corneal plugs at the ends of the two haptics and facilitates a good centration and positioning [16].

Different surgical approaches have already been reported for the implantation of this innovative lens, showing overall encouraging anatomic and refractive outcomes. According to the specific surgical variant, the sclero-corneal plugs can be placed inside scleral pockets, covered by pre-sculpted partial-thickness scleral lamellae, or left beneath the conjunctiva [15, 17].

The purpose of this prospective study is to evaluate the anatomical and functional results of sutureless FIL SSF IOL implantation performed with two novel flapless techniques.

## Methods

### Study design

This multicentric study was carried out at the Unit of Ophthalmology, University of Bologna, Italy and at MicroInvasive Ocular Surgery Center, Lausanne, Switzerland. The study adhered to the tenets of the Declaration of Helsinki and was approved by the Institutional Review Board/Ethics Committee of the local health service in Bologna, Italy (Code CE: 177/2023). Written informed consent was obtained from all subjects included in the study.

In the present study, we included consecutive patients recruited between April 2021 and September 2023 with IOL-bag complex subluxation or phacodonesis, aphakia, IOL luxation in the vitreous chamber, crystalline lens luxation, and IOL-related complications requiring IOL explantation (e.g., IOL opacification, pseudophakic bullous keratopathy). All patients underwent sutureless scleral fixation of FIL SSF intraocular lens using these novel surgical techniques, with a minimum follow-up of 24 months.

Patients underwent either the trocarless or the trocar-assisted technique in a non-systematic fashion, based on the discretion of the surgeons (DI and MDdS) and availability of instrumentation at the time of surgery, without predefined clinical selection criteria. This approach ensured practical applicability while avoiding operator-related allocation bias.

Exclusion criteria were: age < 18 years; history of diabetic retinopathy, age-related macular degeneration, or any ocular condition that could affect study outcomes.

### Clinical assessment

At baseline assessment, a complete medical history regarding all medical therapies and comorbidities was taken from each subject. Patients received a comprehensive ophthalmological evaluation both before and after the surgical intervention. All indications for secondary IOL implantation were recorded. The eye examinations included the assessment of the best corrected distance visual acuity (BCVA) reported in logarithm of the minimum angle of resolution (LogMAR) and the intraocular pressure (IOP) assessed with Goldmann applanation tonometry, slit lamp examination of the anterior segment and indirect fundus ophthalmoscopy. In cases where a clear fundus view was not available, a B-scan ultrasound was performed. The IOL Master 700 (Carl Zeiss Meditec AG, Jena, Germany) was used to perform pre-operative ocular biometry, and all patients underwent an optical coherence tomography (OCT) of the macula both before and after surgery using a spectral domain (SD)-OCT (Heidelberg Spectralis, Heidelberg Engineering, Heidelberg, Germany). Intraoperative and postoperative complications were recorded.

### Lens design

The FIL SSF IOL is a single-piece, foldable, hydrophilic acrylic IOL, specifically designed for sutureless scleral fixation. The self-anchoring to the sclera is achieved by two T-shaped harpoons that project from the closed haptics. A 5° anterior angulation of the haptics with respect to the optic plate is intended both to improve the distance from the iris, thus reducing the risks of pupillary block and of iris chafing, and to guarantee a more physiologic effective lens positioning. The lens can be injected in the eye through a 2.2-mm or a 2.4-mm corneal incision, and it can be grasped with a 23-G or a 25-G forceps. The right unfolding of the lens can be promptly checked by the surgeon through two tiny asymmetric incisions on the haptics, since they would be in a specular location if the lens was upside down. The optic plate is 6.5 mm wide, while the total diameter of the lens is 13.2 mm. The FIL SSF IOL has a UV filter and a H2O content of 25%. The range of spherical dioptric power is between − 5 and + 35 diopters, and toric lenses are also available. The refractive index of the lens is 1.461, while the A-constant is 118.7 [15, 18].

### Flapless intrascleral fixation technique (trocarless)

The surgical procedures were performed by two surgeons (DI, MDS) under local anesthesia. After the sclera was exposed, two marks with a 12-mm Mendez ring (Moria Surgical, Antony, France) along the horizontal meridian at 3 and 9 o’clock were created. A circumferential partial-thickness scleral groove length of 4 mm was sculpted with a pre-calibrated depth knife (Sharpoint™ Surgical Specialties Mexico - restricted depth knives 300 μm) on both sides 2.5 mm from the limbus. A 25-G needle perforated the deep scleral groove at the center. The SSF-IOL was injected through a 2.4 mm-wide corneal tunnel at 11 o’clock hours after the anterior chamber (AC) was filled with cohesive viscoelastic material. As the IOL unfolded out of the cartridge tip in the AC, a 25-G forceps (GRIESHABER^®^ MAXGRIP^®^ Forceps-Alcon-Grieshaber, Fribourg, Switzerland) was introduced into the vitreous chamber through the 3 o’clock hole. The forceps gently grasped the leading T-shaped harpoon and drove it out of the eye through the scleral hole and groove. At this point, the IOL was completely released from the injector cartridge, free to unfold, and both the optic plate and proximal haptic rested in the AC over the iris plane. The forceps were passed through the 9 o’clock scleral hole in the vitreous chamber to grasp and externalize the remaining T-shaped harpoon with the help of a second forceps or hook inserted through a clear corneal incision. The use of an intraoperative OCT-assisted microscope helped confirm the complete intrascleral harpoon implant with no exposure. The conjunctiva was sutured with 8 − 0 Vicryl. Using these maneuvers, the harpoons were buried in the previously created partial-thickness scleral grooves. See Supplemental material video.

### Flapless trocar-assisted intrascleral fixation technique

The surgical procedures were performed by two surgeons (DI, MDS) under local anesthesia. After exposure of the sclera along the horizontal meridian at the 3 and 9 o’clock positions, a circumferential partial-thickness scleral groove length of 4 mm was sculpted with a pre-calibrated depth knife (Sharpoint™ Surgical Specialties Mexico - restricted depth knives 300 μm) on both sides, 2.5 mm from the limbus. The first trocar for the infusion line was inserted inferotemporal, whereas in the middle of the groove, a 25-G trocar was inserted into each groove, and the required intraocular surgical steps were performed. Following viscoelastic injection in the AC, the lens was slowly extruded into the AC. The first harpoon was grasped using forceps, and the trocar was removed from the eye before pulling the harpoon through the sclera and sliding sufficiently far along the shaft of the forceps to avoid hampering the mechanics of the forceps. Subsequently, the harpoon was extruded and positioned within the groove. The same maneuvers were executed for the other harpoon and trocar. See **Supplemental material video**.

### Statistical analysis

Normality was tested using the Shapiro–Wilk test. As the variables were normally distributed, they were reported as mean and standard deviation, and parametric tests were used for statistical analysis. The Chi-square test was used to measure the association between two categorical variables. The paired t-test was used to evaluate the differences in BCVA and IOP before and after surgery. *P* values < 0.05 were considered statistically significant. Statistical analysis was performed using IBM Statistical Package for Social Sciences version 26.

## Results

### Demographic data

A total of 54 eyes of 54 patients were included in the analysis, 24 right eyes (44.4%) and 30 left eyes (55.6%). The mean age of the patients was 74.0 ± 14.2 years (range, 31 − 96), and 40.7% were female. At the baseline assessment, 34 eyes presented with subluxated IOL (62.9%), 11 eyes with dropped cataract in vitreous chamber (20.3%), 3 eyes with IOL dislocated in the vitreous cavity (5.5%), 2 eyes with traumatic aphakia (3.7%), 2 eyes with iris fixated IOL and bullous keratopathy (3.7%), 1 eye with uveitis-glaucoma-hyphema syndrome (1.8%), and 1 eye with IOL opacification and bullous keratopathy (1.8%). The mean BCVA at the baseline assessment was 0.9 ± 0.9 (range 0.2 −5), and the mean IOP was 20.1 ± 9.2 mmHg. The demographic characteristics of the patients are shown in Table 1.

**Table 1:** Demographic and clinical data at baseline assessment

Variable	Patients (*n* = 54)
Age (years), mean ± SD	74.0 ± 14.2
Sex, female, n (%)	22 (40.7%)
BCVA (LogMAR), mean ± SD	0.9 ± 0.9
IOP (mmHg), mean ± SD	20.1 ± 9.2
Etiology of insufficient capsular support	**n (%)**
IOL subluxation –Spontaneous –PEX –Scleral-fixated 3-piece IOL implant –High myopia	34 (62.9%) 24 (70.5%) 6 (17.6%) 2 (5.8%) 2 (5.8%)
Dropped cataract into vitreous chamber	11 (20.3%)
IOL dropped into vitreous	3 (5.5%)
Traumatic aphakia	2 (3.7%)
Iris-fixated IOL + bullous keratopathy	2 (3.7%)
UGH syndrome	1 (1.8%)
IOL opacification + bullous keratopathy	1 (1.8%)

*SD *= Standard Deviation, *BCVA* = Best corrected visual Acuity, *IOP* = Intraocular Pressure, *IOL* = Intraocular Lens, *PEX* = Pseudoexfoliation syndrome, *UGH* = Uveitis-Glaucoma-Hyphema

### Surgical interventions

The mean intra-operative time was 79.9 ± 23.2 min. In 36 eyes (66.7%), FIL SSF IOL implantation was preceded by IOL explantation and complete pars plana vitrectomy (PPV), in 12 cases (22.2%) only complete PPV was performed with FIL SSF IOL implantation, in 4 eyes (7.4%) FIL SSF IOL implantation was preceded by IOL explantation, in 1 eye previously vitrectomized (1.8%) only FIL SSF IOL implantation was performed, and in 1 eye FIL SSF IOL implantation was preceded by IOL explantation, complete PPV and XEN gel stent implantation (1.8%). No intra-operative or early post-operative complications were encountered.

### Trocarless vs. trocar-assisted SSF-IOL scleral implant

A total of 43 eyes (79.6%) underwent flapless intrascleral fixation technique (trocarless), and 11 eyes (20.4%) underwent flapless trocar-assisted SSF-IOL scleral implant. No intra-operative or early post-operative complications were encountered, and no significant differences in the mean intra-operative time were observed between the two groups (78.4 ± 23 min vs. 80.2 ± 24 min, *p* = 0.186).

### Follow-up assessment at 24 months

After 24 months from surgery (mean follow-up 25 ± 1 months), BCVA significantly improved from 0.9 ± 0.9 to 0.4 ± 0.5 LogMAR (*p* < 0.001). The mean value of IOP decreased from 20.1 ± 9.2 to 17.0 ± 3.7 mmHg (*p* = 0.083). Among the 54 eyes included in the study, 7 (12.9%) showed IOP values exceeding 21 mmHg, which were managed with a dorzolamide–timolol fixed combination, and after 24 months from surgery, only one patient presented with values of IOP > 21 mmHg (1.8%). Also, we observed 2 cases of cystoid macular edema developed after a mean of 3 ± 1 months from surgery (3.7%). These patients were managed with topical treatment in both cases, achieving complete resolution at the last follow-up visit. Furthermore, we observed 1 case of FIL SSF IOL tilting detected 4 months from surgery, 1 case of scleral show detected 6 months from surgery, 2 cases of corneal decompensation requiring endothelial keratoplasty, 1 case of retinal detachment after 7 months from surgery, and 1 case of subretinal hemorrhage after surgery. These cases are currently under follow-up. A summary of postoperative complications is reported in Table 2. No significant differences in complication rates were observed between the trocarless and trocar-assisted groups (*p* > 0.05).

**Table 2:** Postoperative complications observed during the 24-month follow-up

Complications	*n* (%)
Increased IOP	7 (12.9%)
Cystoid macular edema	2 (3.7%)
Corneal decompensation	2 (3.7%)
FIL SSF IOL tiltation	1 (1.8%)
Scleral show	1 (1.8%)
Retinal detachment	1 (1.8%)
Subretinal hemorrhage	1 (1.8%)

IOP = Intraocular Pressure; IOL = Intraocular Lens

## Discussion

The management of eyes with poor or absent capsular support still represents an essential challenge for surgeons, and a great variety of alternative solutions for intraocular lens implantation in such cases have been proposed over time, including AC-IOLs, IF-IOLs, and scleral fixated posterior chamber IOLs (PC-IOLs). In 2003, the American Academy of Ophthalmology published a comprehensive literature review about secondary IOL implantation without capsular support [19]. The different IOL models and techniques were compared in terms of safety, efficacy, and complication rates. Based on the available data at that time, the authors concluded that there was insufficient evidence to recommend a specific type of IOL and implantation procedure over any other for patients in whom all options were feasible. A particular emphasis has been put in recent years on PC-IOLs: they indeed could theoretically represent a desirable choice over other solutions, since they seem to provide an optical advantage related to their more natural anatomic positioning and proximity to the nodal point of the eye [20]. A dramatic evolution has occurred concerning techniques for secondary implantation of PC-IOLs. In the 1980 s, Malbran et al. [21] published their pioneering technique for PC-IOL fixation, which, however, was not free from major drawbacks, such as a long learning curve, the need for a large corneal incision and scleral suturing, and the potential complications derived from suturing rupture and erosion. In this sense, another milestone was represented by the advent of sutureless techniques, which shared the off-label use of various 3-piece IOL models. Concerns arose about the long-term stability of these IOLs, since their haptics were not intended for trans-scleral passage and fixation [15].

Given this scenario, the advent of the FIL SSF IOL was a significant step forward, both because it was specifically conceived for scleral fixation and because it could be implanted without the need for sutures [22].

Since the lens made its market debut, several surgical variants have already been described for its implantation. Among others, Fiore et al. [23] described a technique that involved the creation of two straight 2.5-mm radial incisions at the 0° and 180° axes, after conjunctival peritomy. Within each incision, the sclera was dissected to generate two opposing pockets on each side of the incisions, where the externalized plugs of the lens could be placed.

Rossi et al. [24] reported a different approach, sculpting two 3.5 × 3.5 mm partial-thickness scleral lamellae at 3- and 9-o’clock positions. The deep scleral lamellae were then perforated with a 25-G needle at 1.5 mm from the limbus, to create the holes for the trans-scleral passage of the IOL harpoons. Caporossi et al. [25] published another surgical variation in which the two vitrectomy ports were employed as the fixation sites for the lens plugs, which were then allocated inside two opposite scleral pockets.

Despite overall good safety and efficacy outcomes, none of the techniques reported in the literature were completely free from potential complications, including post-operative hypotony, IOL opacification [26], haptic exposure under the conjunctiva [27], thinning of the scleral flaps, and late retinal tears or retinal detachment.

Also, previously published techniques can present inter-operator and intra-operator variability, and crucial maneuvers such as scleral flap sculpture or radial incision with two opposite pockets are related to the surgeon.

The creation of scleral flaps can be one of the crucial steps of the SSF-IOL implant, with the flap being too thin or too thick, or inadvertent perforation with subsequent loss of the anchorage site.

In the present study, we reported the outcome data from a cohort of patients who underwent the sutureless scleral fixation of a FIL SSF IOL according to a novel modified surgical technique.

Visual acuity showed a significant modification at 24-month follow-up from the baseline, improving from a mean of 0.9 ± 0.9 to 0.4 ± 0.5 LogMAR (*p* < 0.001). These results are in good agreement with those of previously published works [24, 27, 28], even though the different follow-up times and the heterogeneity of surgical indications for secondary IOL implantation (e.g., post-traumatic aphakia) cannot be overlooked when evaluating the comparability of the functional outcomes. Moreover, none of the patients in our study experienced a decrease in BCVA, except those who experienced major complications. Previous studies concerning different techniques for PC-IOL sutureless scleral fixation reported various rates of eyesight deterioration [29, 30]. One of the most innovative aspects of this surgical variant was the reduced manipulation of the scleral tissue, since the sculpting of lamellar scleral flaps and the creation of scleral pockets were substituted by the creation of two circumferential partial-thickness scleral grooves, located 180° away from each other.

This approach could bring some advantages: among others, a shorter surgical time for IOL implantation, and a potentially shorter learning curve. In contrast to other studies [25], no cases of post-operative hypotony were encountered. This result is likely attributable to the optimal fitting of the 0.3 mm diameter of the T-shaped harpoon into the 25-G sclerotomy, acting as a plug. Moreover, the corneal incision and the pars plana vitrectomy sclerotomies were carefully sutured.

It is also noteworthy that the use of a pre-calibrated depth knife to make the scleral grooves represents, where available, a way to standardize the technique – by commuting it in a non-operator dependent manner - and, at the same time, a crucial safety tool in the hands of the surgeon to avoid inadvertent full-thickness scleral penetration. According to Buckhurst et al., the scleral wall between 2 and 3 mm from the limbus is more than 600 microns thick in all quadrants [31], which allows for a safe implant with a pre-calibrated depth of 300 microns. No cases of post-operative leakage from the wounds or hypotony were, indeed, observed in our cohort.

As long-term safety issues are concerned, a particular emphasis must be placed on the risks of conjunctival erosion and/or infections that could derive from the direct exposure of the lens haptic to the conjunctiva. In a previous study, Veronese et al. [32] described a different procedure: the externalized sclero-corneal plugs were deliberately left beneath the conjunctiva. Despite the ease and rapidity of the procedure, the relatively short follow-up time in that study might have prevented the recognition of later complications.

**Fig. 1:**
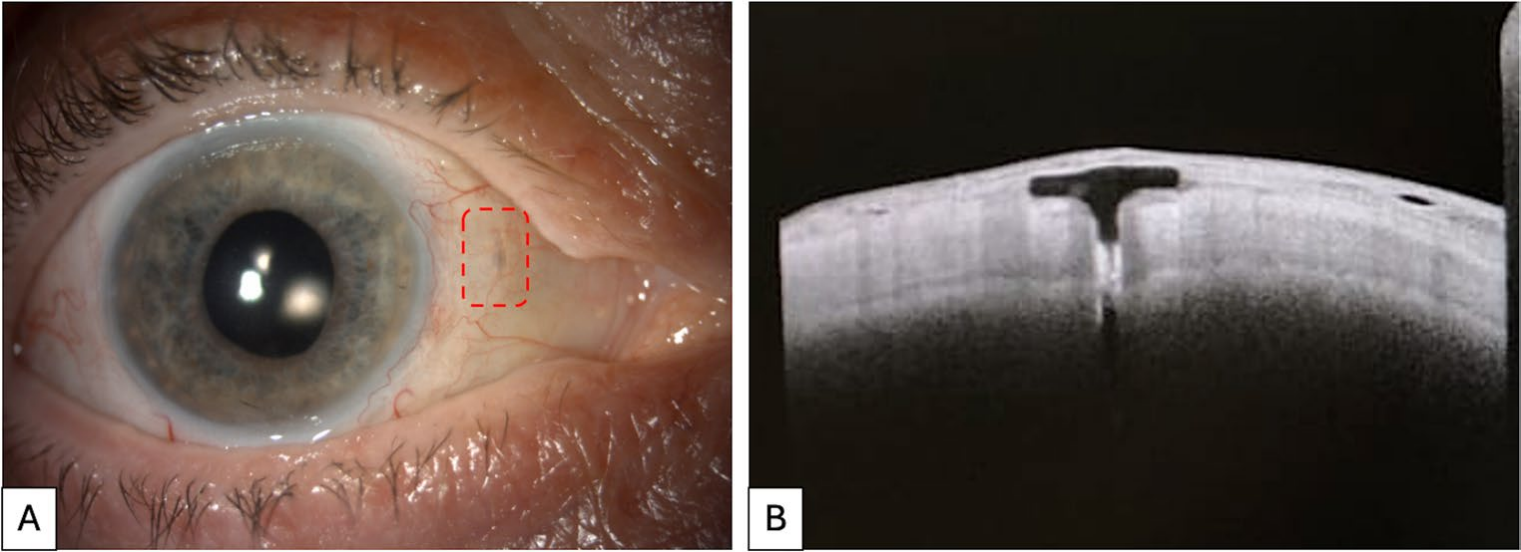
(**A**) Clinical appearance of FIL SSF IOL after flapless scleral fixation in a right eye at 24-month follow-up; the harpoon at the 3 o’clock position is visible through the conjunctiva, lying inside the circumferential scleral groove and surrounded by a dotted outline (**B**) Anterior segment optical coherence tomography of the same eye shows correct allocation of the T-shaped harpoon inside the sclera

In our study, the depth of the scleral grooves was sufficient in every patient to prevent direct contact between the lens plugs and the conjunctiva, as demonstrated by post-operative AS-OCT (see Fig. 1**)**.

Furthermore, there was no evidence of harpoon exposure or conjunctival erosion, at least until the 24-month follow-up visit. The external part of the harpoon is, indeed, 200 microns thick and can be hosted easily by a 300 microns deep scleral groove. The circumferential orientation of the grooves is another noteworthy aspect of this technique, since they can better fit the architecture of the harpoons, which are also circumferentially oriented.

On the other hand, in our cohort we observed an increase in IOP above 21 mmHg after surgery in a small proportion of patients, who were successfully managed with a combination of topical IOP-lowering drugs until the last follow-up visit. Despite what was reported with other fixation techniques [12, 14], no cases of iris capture were encountered in our cohort. This result is likely attributable to the self-centration and the good positioning due to the firm fixation of the lens, even if further studies aimed at investigating the IOL tilting and the distance of the lens from the cornea should be helpful to confirm these hypotheses.

Our study likely suffered the pitfalls that could derive from the lack of a direct comparison with a control group undergoing implantation of FIL SSF IOL according to a different surgical technique.

Also, we cannot exclude that a longer follow-up time could reveal complications occurring a long time after surgery, as it has been observed for complications relative to other techniques, that were noted many years after the surgical procedure (e.g., suture erosion).

## Conclusions

The scleral fixation of the FIL SSF intraocular lens could represent a valuable option for managing cases with insufficient or absent capsular support. Based on the findings of the present study, this novel modified flapless technique may represent a safe and effective approach for sutureless implantation of the FIL SSF IOL, as it combines the distinctive advantages of this innovative lens—such as controlled intraocular unfolding, firm self-anchoring to the sclera, and reliable self-centration—with minimal tissue manipulation, a potentially low complication rate, and a shorter learning curve .

## Supplementary Information

Below is the link to the electronic supplementary material.

Supplementary Material 1 (MP4 94.7 MB)

## Data Availability

The data sets used and/or analyzed during the current study are available from the corresponding author on reasonable request.

## Abbreviations

AC: Anterior Chamber
AC-IOL: Anterior Chamber Intraocular Lens
AS-OCT: Anterior Segment Optical Coherence Tomography
BCVA: Best Corrected Visual Acuity
IF-IOL: Iris-Fixated Intraocular Lens
IOL: Intraocular Lens
IOP: Intraocular Pressure
OCT: Optical Coherence Tomography
PC-IOL: Posterior Chamber Intraocular Lens
PPV: Pars Plana Vitrectomy
SD-OCT: Spectral Domain Optical Coherence Tomography
SF-IOL: Scleral-Fixated Intraocular Lens
UV: Ultraviolet
